# Three-Dimensional Tissue Strain Measurement Using a Row–Column Array During Biaxial Testing of Excised Ventricular Porcine Myocardium

**DOI:** 10.1016/j.ultrasmedbio.2025.05.007

**Published:** 2025-06-13

**Authors:** Xavier Navy, Zhiyu Sheng, Kang Kim, John M. Cormack

**Affiliations:** a Department of Electrical Technology, Houston Community College, Houston, TX, USA; b Division of Cardiology, Department of Medicine, University of Pittsburgh, Pittsburgh, PA, USA; c Vascular Medicine Institute, University of Pittsburgh and University of Pittsburgh Medical Center, Pittsburgh, PA, USA; d Department of Bioengineering, University of Pittsburgh, Pittsburgh, PA, USA

## Abstract

**Objective:**

To implement and validate a 3D volume imaging sequence and 3D strain estimation procedure for enhanced biaxial mechanical testing of excised ventricular myocardium.

**Methods:**

One specimen of right and one of left ventricular excised porcine myocardium were tested using dual-loading protocol quasistatic biaxial mechanical testing. During biaxial testing, volume ultrasound (US) images were acquired using a row–column addressed array probe using a synthetic aperture imaging sequence. Volume US images were used to compute tissue deformation using 3D correlation-based US speckle tracking. US-derived tissue strains were validated against repeated measurements using conventional optical camera imaging of the specimen surface deformation.

**Results:**

Speckle tracking yielded high-fidelity maps of tissue deformation in 3D throughout the entire sample volume. US-derived tissue strain is in good agreement with ground-truth camera-derived surface strain measurements (root mean square error is 1.6% strain).

**Conclusion:**

The 3D full-thickness strain measurement with US imaging is accurate and can enhance biomechanical insights from biaxial experimentation, especially in large tissues such as porcine and human myocardium where assumptions of plane stress and incompressibility may not apply.

## Introduction

Biaxial mechanical testing (BMT) is a popular benchtop experimental technique for determining the anisotropic mechanical properties of excised soft tissues [1], including ventricular myocardium, [2,3] bladder [4], and vascular [5] applications. In current practice, high-fidelity force measurements are coupled with tissue deformation measurements by the tracking of superficial markers in optical camera images. Thus the measurement technique is not sensitive to out-of-plane motion and deformation, that is, perpendicular to the sample surface, and the inverse problem of determining tissue mechanical properties must be made tractable by using assumptions such as plane stress and incompressibility that may not apply in all cases [6–8]. Other imaging techniques such as optical coherence tomography or multiphoton microscopy can image below the tissue surface, but only up to depths of less than 1 mm without the use of mechanical property-altering tissue clearing [9,10].

Previous studies of coupling ultrasound (US) imaging with biaxial mechanical testing of excised myocardium enabled full-thickness measurement of 3D displacement and strain by using linear [11] or rotational [8] scans of linear US imaging arrays along with 3D US speckle tracking (3DUST) techniques [12]. Mechanical scanning of the US imaging array resulted in long data acquisition times, thereby limiting testing to quasistatic deformations and limiting the number of deformed configurations that could be produced during a reasonable testing duration.

In this letter, we introduce and validate 3D strain measurement during biaxial testing of ventricular myocardium without mechanical scanning by acquiring volumetric 3D US images using a row–column addressed imaging array (RCA) and synthetic aperture beamforming [13]. We used 3DUST to obtain displacement and strain throughout the specimen volume. Strain calculated by 3D US imaging is validated against strain measured on the surface using conventional camera imaging.

## Methods

### Tissue preparation and biaxial testing

The right and left ventricular free walls of a fresh frozen porcine heart were removed from the septum, and 20 × 20 mm^2^ square sections were excised following the procedure in Ref. 8. Briefly, the right ventricular (RV) sample was excised from near the tricuspid valve annulus oriented such that the X axis of the sample was parallel to the apex–outflow tract direction. The left ventricular (LV) sample was excised from near the mitral valve annulus and oriented with rotation approximately 45° to the mitral valve annulus. The RV sample was left at full thickness (approximately 8 mm) for testing, and the LV sample was thinned to approximately 5 mm thickness including the epicardium.

**Figure 1.**
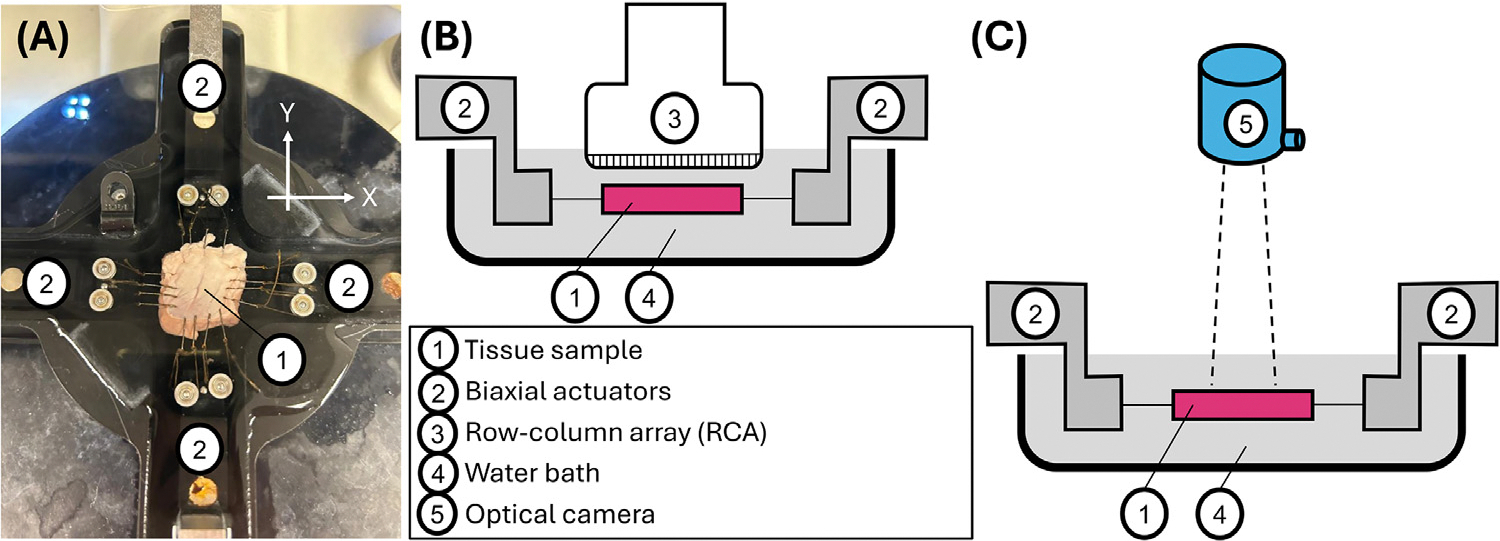
(A) Top view photograph of an excised square sample of right ventricular myocardium loaded into the BMT device. Schematics of measurement configurations using (B) 3D ultrasound examination, and (C) optical camera imaging.

Each tissue sample was loaded into the BMT machine (BioTester 5000, CellScale, Ontario, Canada) using fishhooks and pulleys to reduce shear loading (Fig. 1A) [1,6]. The sample was submerged in deionized water at approximately room temperature. Two-protocol displacement-controlled quasistatic biaxial stretching was used [8,11] in which the actuator displacements in the two loading protocols had ratios of 1:1 and 1:2 (X:Y), respectively. Preconditioning and re-tensioning procedures [8] were followed before the testing cycles to account for tissue damage that occurred during preconditioning. Three stretched configurations were obtained for each of the two stretching protocols (six stretched configurations total per sample). The maximum actuator displacement in both test protocols was approximately 4 mm.

### US volume imaging and 3D strain calculation

An RCA (RC6gV, Vermon SA, Tours, France; 6.0 MHz center frequency, 256 channels, 25.4 × 25.4 mm^2^ footprint) was poised above the tissue before BMT (Fig. 1B) with lateral and elevational axes aligned with the BioTester’s X and Y actuators. US imaging during BMT was controlled by a programmable research US imaging platform (Vantage 256, Verasonics, Kirkland, WA, USA).

Volume images were acquired in the reference configuration (after preconditioning and re-tensioning) and while the sample was held in each of the stretched configurations (seven total volume images per sample tested). Imaging was performed with a synthetic aperture approach based on Ref. 13. Briefly, the imaging sequence was to transmit from each individual element sequentially. Immediately after each row (column) element transmission, all of the column (row) elements record the echo signals simultaneously. A custom delay-and-sum beamforming routine was used to reconstruct the US volume image. The reconstructed volumes had dimensions of 25.4 × 25.4 mm in the X-Y plane and a depth of 17 mm with a voxel size of 0.2 × 0.2 × 0.12 mm, thus capturing the entire tissue sample volume.

The US image signal used for 3DUST was the B mode brightness (in decibel units). 3DUST of each voxel in the imaging volume is not computationally feasible, therefore 3DUST was performed in 13 X-Z (lateral–axial) planes spaced 1.5 mm apart [8,12]. A 3 × 3 × 3 mm cubic kernel and a search distance of 1.5 mm in each direction was used for 3DUST. A median filter was applied to each computed X-Z cross-section of each displacement component with a kernel size of 1.0 × 0.6 mm. Tissue motion in three dimensions throughout the tissue volume was determined in this way between each deformed configuration of each stretch protocol.

The trajectory x(X) of a particle that began at $X=|X,Y,Z{|}^{T}$ was obtained by accumulating displacements measured from 3DUST. The positions $x_{i}$ of particles in configuration i were found iteratively from

(1) $$x_{i}=x_{i-1}+{\bar{u}}_{i}\left(x_{i-}1\right),\;i=1,2,3,$$

where ${\bar{u}}_{i}\left(x_{i-1}\right)$ are the displacements measured by 3DUST between deformed configurations and $x_{0}=X$. Interpolation using Delaunay triangulation (implemented with scatteredInterpolant in Matlab; MathWorks, Natick, MA, USA) is used to estimate ${\bar{u}}_{i}\left(x_{i-1}\right)$ for calculations if the deformed position $x_{i-1}$ does not fall onto the grid on which 3DUST was performed. The total displacement throughout a deformation protocol is then $u_{i}(X)=x_{i}(X)-X$. Displacement accumulation was performed for tissue particles that had 3DUST normalized correlation coefficients greater than 0.35 and B-mode brightness greater than −50 dB, respectively, during the loading protocol.

The displacement gradient H=∂u/∂X was estimated by fitting a linear model to the total displacement u(X) in each stretched configuration obtained from the accumulation. The region of fit was a rectangular parallelepiped volume with 14 mm on each lateral (X) and elevational (Y) sides, and included the entire tissue sample thickness in the axial (Z) direction as identified in the US volume image of the reference configuration. The coefficients of the linear models fit to each displacement component are the elements of H:

(2) $$u_{X}\approx H_{11}X+H_{12}Y+H_{13}Z+\text{const.,}$$

(3) $$u_{Y}\approx H_{21}X+H_{22}Y+H_{23}Z+\text{const.,}$$

(4) $$u_{Z}\approx H_{31}X+H_{32}Y+H_{33}Z+\text{const..}$$

The deformation gradient tensor was calculated as F=I+H, where I is the identity tensor. The finite strain tensor E was obtained from the displacement gradient using

(5) $$E_{ij}=\frac{1}{2}\left(H_{ij}+H_{ji}+H_{ki}H_{kj}\right),$$

where the index summation convention is used. Thus, the six strain components were computed for each deformed configuration of the tissue sample, including the three in-plane ($E_{XX}$, $E_{YY}$, and $E_{XY}$) and three out-of-plane ($E_{YZ}$, $E_{ZY}$, and $E_{ZZ}$) components.

### Validation of strain calculation

After biaxial stretching while imaging with the RCA, the RCA was removed and the optical camera was put in place aimed at the center of the tissue surface (Fig. 1C) [3,8,11]. The test cycles were repeated while the camera captured images at a rate of 1 Hz. Displacement of the tissue surface in two dimensions was estimated using the Labjoy software (CellScale) [8], and in-plane strain $E_{XX},E_{YY},E_{XY}$ computed using eqns. (2) through (5). Out-of-plane strain components are not accessible to camera imaging.

### Stress calculation

The second Piola–Kirchhoff stress $S={PF}^{-1}$, where P is the first Piola–Kirchhoff stress, was obtained by assuming plane stress and using the formulation given by Ref. 6:

(6) $$\begin{array}{l} \left[\begin{array}{cccc} 1 & 1 & 0 & 0\\ 0 & 0 & 1 & 1\\ H_{21} & 1+H_{22} & -\left(1+H_{11}\right) & -H_{22}\\ 1+H_{11} & H_{12} & H_{21} & 1+H_{22} \end{array}\right]\left[\begin{array}{c} P_{11}\\ P_{12}\\ P_{21}\\ P_{22} \end{array}\right]\\ \;=\frac{1}{LT}\left[\begin{array}{c} f_{1}\\ f_{2}\\ 0\\ \left(1+H_{11}\right)f_{1}+\left(1+H_{22}\right)f_{2} \end{array}\right], \end{array}$$

where L is the sample width (approximately 20 mm, measured before testing), T is the sample thickness in the reference configuration (measured from US images), and $f_{1}$ and $f_{2}$ are the forces measured by the BioTester load cells parallel to the X and Y directions, respectively.

## Results

**Figure 2.**
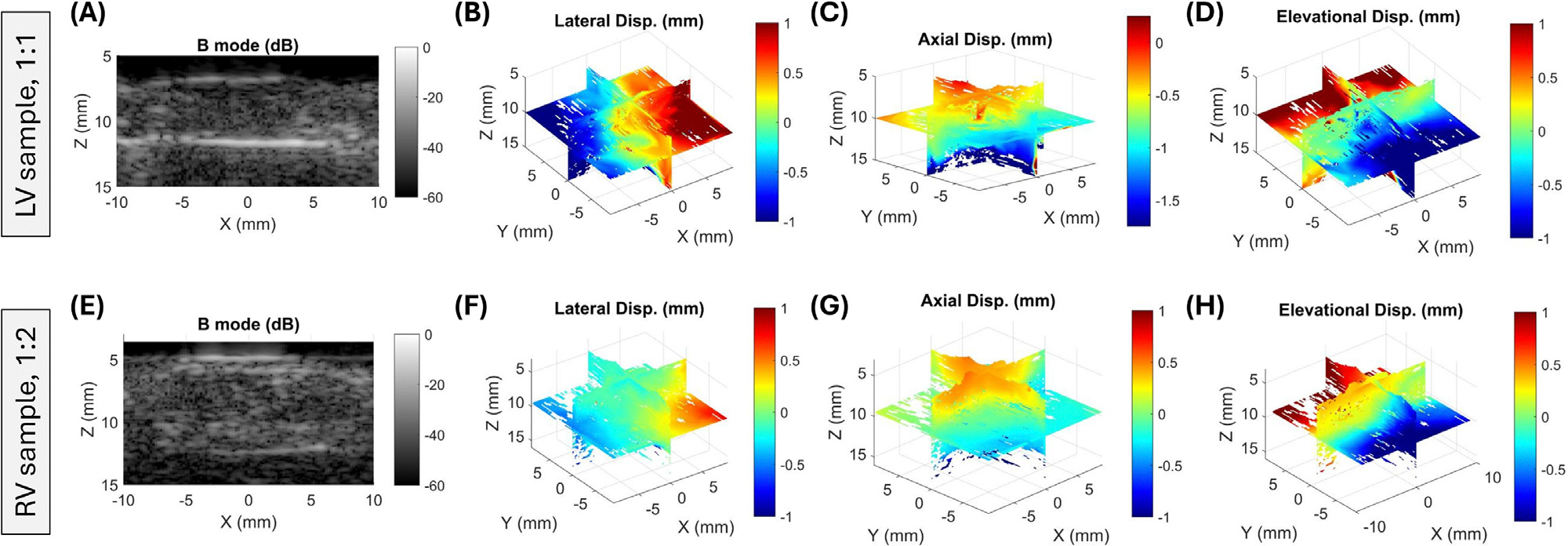
X-Z cross-section of the 3D ultrasound image and triplane views of displacement vector components throughout the specimen volume for (A–D) the maximum stretch for 1:1 aspect ratio protocol of the LV specimen, and (E–H) the maximum stretch for 1:2 aspect ratio protocol of the right ventricular specimen. Lateral, axial, and elevational displacement refer to displacement in the X,Z, and Y directions, respectively.

The displacement field vector was well captured by 3DUST of the volume US images throughout the tissue volume. The mean of $R^{2}$ values for the linear fits eqns. (2) through (4) of all deformed configurations were 0.78, indicating a high-fidelity displacement measurement. Displacement vector fields corresponding to the maximum stretch of the LV sample during the 1:1 (approximately equibiaxial) protocol and the RV sample during the 1:2 protocol are shown in Fig. 2.

**Figure 3.**
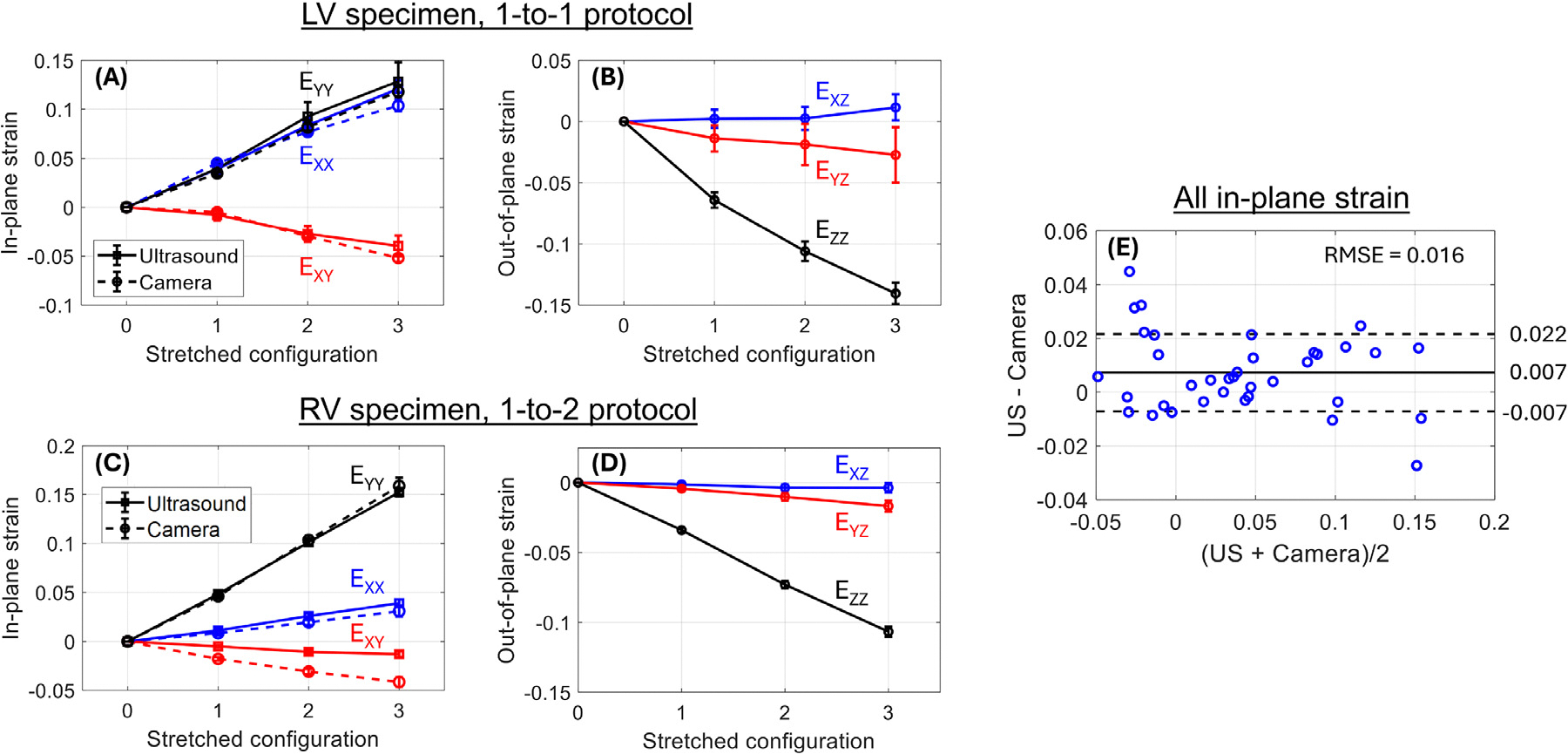
(A–D) In-plane and out-of-plane strain components computed from 3D ultrasound images (*solid curves*) for the left ventricular specimen under 1:1 loading (A, B) and the right ventricular specimen under 1:2 loading (C, D). *Dashed curves* in (A) and (C) are optical camera-derived strain (ground truth). (E) Bland–Altman representation of all in-plane strain measurements $E_{XX},\;E_{YY}$, and $E_{XY}$ (*blue circles*); *solid and dashed horizontal lines* are mean difference and ± one standard deviation of difference. Root mean square error is 1.6% strain.

In-plane strain $E_{XX},\;E_{YY}$, and $E_{XY}$ measured by US is in overall good agreement with ground truth optical camera-derived strain (Figs. 3A and 3C). The mean error of all 36 in-plane strain measurements covering both samples and each deformed configuration per loading protocol is 0.7% strain with a standard deviation of 1.4% strain (Fig. 3E). The root mean square error is 1.6% strain, similar to our previous study [8]. The statistically significant error bias (*p* = 0.005) possibly resulted from tissue damage sustained during the first test cycles such that the tissue stretched less for the same actuator displacements during the repeated cycles that were observed by the camera. In addition, 3DUST enables the measurement of out-of-plane strain $E_{XZ},\;E_{YZ}$, and $E_{ZZ}$ with low uncertainty (Figs. 3B and 3D).

**Figure 4.**
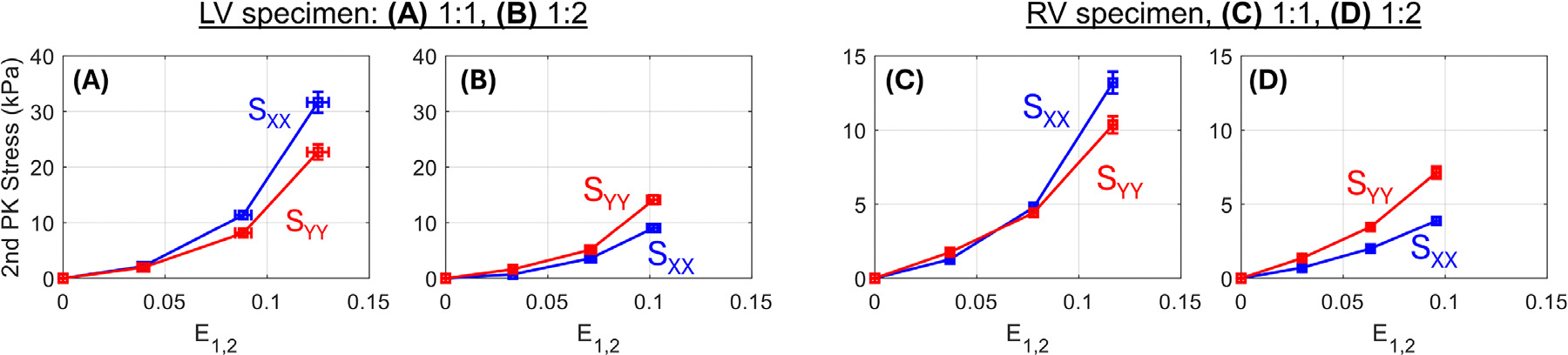
The second Piola–Kirchhoff stresses $S_{XX}$ and $S_{YY}$ versus mean positive principal strain $E_{1,2}$ from 3DUST for the (A) LV specimen in a 1:1 stretch protocol, (B) LV in a 1:2 protocol, (C) RV specimen in a 1:1 protocol, and (D) RV in the 1:2 protocol. Curves are straight lines meant to guide the eye between measured data points.

Measured stress–strain curves (Fig. 4) reveal similar stiffness, non-linearity, and anisotropy as in previous studies of porcine ventricular myocardium [8,15]. The LV specimen in this study exhibited approximately double the stiffness of the RV sample.

## Discussion

US volume imaging with an RCA enables accurate calculation of tissue strain during BMT, including 3D strain throughout the full thickness of the myocardium samples, offering improved measurement over both conventional optical camera-based techniques and advanced optical techniques like optical coherence tomography and multiphoton microscopy. Accuracy was quantified by agreement between US and camera-derived surface strains. A notable discrepancy is in the $E_{XY}$ curves of Fig. 3C, which is possibly due to millimeter-scale heterogeneity in the deformation of the RV surface that includes the right coronary artery and a thin surficial fat layer. This heterogeneity can result in disagreement with US-derived strain that accounts for deformation of the entire tissue volume [8]. The discrepancy is an outlier in the present study (Fig. 3E).

Volume imaging further enables faster 3D imaging than past approaches that used a mechanical scan of a linear array transducer [8,11]. Faster volume image acquisition can enable more robust biomechanical testing by allowing for the observation of a larger number of deformed configurations in a reasonable testing duration. High frame rate volume imaging (up to 100 Hz for our sequence) can be used to study dynamic deformations and myocardium viscoelasticity, which are of recent interest [14].

The RCA probe used in this study, although commercially available, is not ideally suited for 3DUST in the context of BMT. The ideal volume imaging array would have a smaller footprint closer to the lateral-elevational dimension of the samples (20 × 20 mm), and higher US frequency (e.g., 18 MHz or 30 MHz used in previous studies [8,11]) for a more optimal tradeoff of spatial resolution versus imaging depth.

The major limitation of the approach is processing time. The 3DUST calculation for one deformed configuration required several hours despite limiting calculation to 13 X-Z planes, compared with several seconds for computation of surface strain from camera images. Faster cross-correlation algorithms can improve 3DUST computation times [16].

## Conclusion

A method was implemented and validated for measurement of full-volume 3D tissue deformation and six-component tissue strain using US volume imaging with a row–column array during BMT of porcine ventricular myocardium. The method can improve biomechanical testing of thick myocardial specimens where assumptions of plane stress or incompressibility may not apply.

## Data Availability

All data are available from the authors upon reasonable request.

## References

[1] Sacks MS. Biaxial mechanical evaluation of planar biological materials. J Elasticity 2000;61:199–246.

[2] Hill MR, Simon MA, Valdez-Jasso D, Zhang W, Champion HC, Sacks MS. Structural and mechanical adaptations of right ventricle free wall myocardium to pressure overload. Ann Biomed Engr 2014;42:2451–65. 10.1007/s10439-014-1096-3 PMC4241140 25164124

[3] Sharifi Kia D, Benza E, Bachman TN, Tushak C, Kim K, Simon MA. Angiotensin receptor-neprilysin inhibition attenuates right ventricular remodeling in pulmonary hypertension. J Am Heart Assoc 2020;9:e015708. 32552157 10.1161/JAHA.119.015708 PMC7670537

[4] Cheng F, Watton PN, Pederzani G, Kurobe M, Takaoka E-i, Chapple C, A constrained mixture-micturition-growth (CMMG) model of the urinary bladder: application to partial bladder outlet obstruction (BOO). J Mech Behav Biomed Mat 2022;134:105337. 10.1016/j.jmbbm.2022.105337 PMC9835014 35863296

[5] Tamimi E, Ardila DC, Haskett DG, Doetschman T, Slepian MJ, Keller RS, Biomechanical comparison of glutaraldehyde-crosslinked gelatin fibrinogen electrospum scaffolds to porcine coronary arteries. J Biomech Engr 2016;138:011001. 10.1115/1.4031847 PMC4844094 26501189

[6] Sommer G, Ch Haspinger D, Andra M, Sacherer M, Viertler C, Regitnig P, Quantification of shear deformations and corresponding stresses in the biaxially tested human myocardium. Ann Biomed Engr 2015;43:2334–48. 10.1007/s10439-015-1281-z 25707595

[7] McEnvoy E, Holzapfel GA, McGarry P. Compressibility and anisotropy of the ventricular myocardium: experimental analysis and microstructural modeling. J Biomech Engr 2018;140:081004. 10.1115/1.4039947 30003247

[8] Cormack JM, Simon MA, Kim K. Backscatter tensor imaging and 3D speckle tracking for simultaneous ex vivo structure and deformation measurement of myocardium. Ultrasound Med Biol 2023;49:1238–47. 36858914 10.1016/j.ultrasmedbio.2023.01.009 PMC10050135

[9] Aumann S, Donner S, Fischer J, Muller F. Optical coherence tomography (OCT): Principle and technical realization. In: Bille JF, editor. High resolution imaging in microscopy and ophthalmology. Cham: Springer; 2019. 32091846

[10] Theer P, Denk W. On the fundamental imaging-depth limit in two-photon microscopy. J Opt Soc Am A 2006;23:3139–49. 10.1364/josaa.23.003139 17106469

[11] Yap CH, Park DW, Dutta D, Simon MA, Kim K. Methods for using 3-D ultrasound speckle tracking in biaxial mechanical testing of biological tissue samples. Ultrasound Med Biol 2015;41:1029–42. 25616585 10.1016/j.ultrasmedbio.2014.10.021 PMC4346411

[12] Chen X, Xie H, Erkamp R, Kim K, Jia C, Rubin JM, O’Donnell M. 3-D correlation-based speckle tracking. Ultrasonic Imaging 2005;27:21–36. 16003924 10.1177/016173460502700102

[13] Jensen JA, Schou M, Jrgensen LT, Tomov BG, Stuart MB, Traberg MS, Anatomic and functional imaging using row–column arrays. IEEE Transactions on Ultrasonics, Ferroelectrics, and Frequency Control 2022;69:2722–38. 35839193 10.1109/TUFFC.2022.3191391

[14] Nordsletten D, Capilnasiu A, Zhang W, Wittgenstein A, Hasjicharalambous M, Sommer G, A viscoelastic model for human myocardium. Acta Biomat 2021;135:441–57. 10.1016/j.actbio.2021.08.036 34487858

[15] Nemavhola F Study of biaxial mechanical properties of the passive pig heart: material characterisation and categorisation of regional differences. Int J Mech Mat Engr 2020;16:14.

[16] Luo J, Konofagou E. A fast normalized cross-correlation calculation method for motion estimation. IEEE Trans Ultrason Ferroelectr Freq Control 2010;57:1347–57. 20529710 10.1109/TUFFC.2010.1554 PMC4123965

